# Minimally invasive mitral and tricuspid valve surgery using central arterial perfusion

**DOI:** 10.1186/1749-8090-8-S1-O293

**Published:** 2013-09-11

**Authors:** T Tedoriya, M Fukuzumi, R Okano

**Affiliations:** 1 Cardiovascular Surgery, Ageo Central General Hospital, Ageo, Japan

## Background

Minimally invasive cardiac surgery (MICS) is becoming popular at specialized centers with port-access technique. However, some patients who have atherosclerotic lesions in the arteries would have non-negligible risk with peripheral perfusion. The purpose of this retrospective study was to clarify the necessity of the central aortic perfusion technique in MICS.

## Methods

Our strategy for CPB management for MICS was as below: we evaluated atherosclerotic lesions in MICS candidates with CT examination in order to investigate condition of inner cavity of the aorta or arteries, especially the presence of intramural thrombus. In cases of no lesion of possible arteriosclerosis, we selected peripheral perfusion. Otherwise, we used central arterial perfusion with direct cannulation on the ascending aorta. For venous drainage, two-staged venous cannula via the femoral vein and additional SVC cannulation was applied.

From November 2007 to April 2013, we had 81 (32 females, mean age of 68.3 +/- 11.8 y.o.) for MVP or MVR with/without TAP or Maze including 6 redo cases. After evaluation for atherosclerosis, we had applied peripheral perfusion in 9 cases (11%). The other cases were required the ascending aortic cannulation.

## Results

All procedures were accomplished as scheduled without conversion to conventional approach. Aortic cross clamping time was 129 +/- 47 min., and ECC time was 172 +/- 47 min. We had no mortality and no cerebral infarction nor any other perfusion problem in the central arterial perfusion. However, one case with redo-MVR using central perfusion had bleeding problem on the site of the aortic cannulation, required on the second CPB. One emergency redo case with the left subclavian artery perfusion had aortic dissection and died with LOS.

## Conclusion

The central perfusion technique is an important and indispensable option for MICS especially for atherosclerosis in order to prevent from any complications regarding cardiopulmonary bypass management.

